# Extracorporeal shock wave therapy versus local corticosteroid injection for the treatment of carpal tunnel syndrome: a meta-analysis

**DOI:** 10.1186/s13018-020-02082-x

**Published:** 2020-11-23

**Authors:** Wenhao Li, Chunke Dong, Hongyu Wei, Zhencheng Xiong, Liubo Zhang, Jun Zhou, Yanlei Wang, Jipeng Song, Mingsheng Tan

**Affiliations:** 1 Beijing University of Chinese Medicine, Beijing, 100029 China; 2 Department of Orthopaedic Surgery, China-Japan Friendship Hospital, Beijing, 100029 China; 3 Institute of Medical Technology, Peking University Health Science Center, Peking University Third Hospital, Beijing, 100089 China; 4 Graduate School of Peking Union Medical College, Chinese Academy of Medical Sciences, Beijing, 100730 China

**Keywords:** Carpal tunnel syndrome, Extracorporeal shock wave, Local corticosteroid injection, Meta-analysis

## Abstract

**Background:**

Many studies have demonstrated the effectiveness of extracorporeal shock wave therapy (ESWT) and local corticosteroid injection (LCI) for the treatment of carpal tunnel syndrome (CTS), and some studies showed that the effect of ESWT was superior to LCI. We performed this meta-analysis to compare the clinical effects across the two therapies.

**Methods:**

Relevant randomized controlled trials (RCTs) comparing ESWT and LCI for the treatment of CTS were searched in electronic database. The Cochrane risk bias tool was used for quality assessment. After data extraction and quality assessment of the included studies, a meta-analysis was performed using RevMan 5.3 software. Mean differences (MDs), odds ratios (ORs), and 95% confidence intervals (CIs) were analyzed. The protocol for this systematic review was registered on INPLASY (202080025) and is available in full on the inplasy.com (10.37766/inplasy2020.8.0025)

**Results:**

A total of 5 RCT studies with 204 patients were included from the electronic database. The meta-analysis results showed that two therapies were not significantly different in terms of visual analog scale (VAS) score (*P* = 0.65), Boston Carpal Tunnel Questionnaire (BQ) score (*P* = 0.14), sensory distal latency (*P* = 0.66), and nerve conduction velocity (NCV) of the sensory nerve (*P* = 0.06). There were significant differences between the results of motor distal latency (*P* < 0.0001), compound muscle action potential (CMAP) amplitude (*P* < 0.00001), and sensory nerve action potential (SNAP) amplitude (*P* = 0.004).

**Conclusions:**

In terms of pain relief and function improvement, the effects of ESWT and LCI are not significantly different. In terms of electrophysiological parameters, LCI has a stronger effect on shortening motor distal latency; ESWT is superior to LCI in improving action potential amplitude. ESWT is a noninvasive treatment with fewer complications and greater patient safety. In light of the heterogeneity and limitations, these conclusions require further research for definitive conclusions to be drawn.

## Introduction

Carpal tunnel syndrome (CTS) is the most common and widely studied nerve entrapment syndrome [1], accounting for approximately 90% of all compressive neuropathies [2]. It is caused by compression of the median nerve while it passes through the carpal tunnel, a limited space. Inflammation, edema, tendon spasm, hormone level changes, and physical activity are all associated with increased nerve compression, causing pain and numbness. In severe cases, muscle weakness in muscles innervated by the median nerve may occur [3]. Based on differing diagnostic criteria, the reported prevalence and incidence of CTS can vary greatly. The use of clinical criteria in diagnosis is more common than the use of electrophysiological criteria. It is generally estimated that 10% of people suffer from CTS at some point in their lives [3]. Additional studies have shown that the incidence and prevalence in middle-aged populations are 0.125–1% and 5–16%, respectively. Various risk factors reportedly include high body mass index, female gender, age, and pregnancy, among others [2]. CTS is also recognized as one of the most prevalent occupational health injuries, particularly in industries where work involves high-intensity, repetitive use of the wrists, and vibration tools. The incidence rate in workers employed in such industries is approximately 5% [2]. Treatment is usually organized into conservative treatment and surgical treatment. The overall therapeutic goals are generally to relieve symptoms, improve function, and prevent further nerve damage. More common treatment methodologies include local corticosteroid injection (LCI) and carpal tunnel release surgery [4].

Extracorporeal shock wave therapy (ESWT) was initially used clinically as a lithotripsy method to destroy calcified deposits in the body, especially stones in the kidney, bile duct, and salivary gland ducts. Over the past 30 years, this technology has been increasingly applied to various musculoskeletal diseases, such as shoulder calcified tendinitis, delayed fracture healing, and others [5]. Many studies, including some randomized controlled trials (RCTs) that have demonstrated the effectiveness of ESWT for the treatment of CTS, and the effect of ESWT is superior to LCI [6–15]. We performed this meta-analysis of related RCTs to compare the efficacy of ESWT and LCI to provide greater evidence for clinical decision-making.

## Methods

This meta-analysis was performed in accordance with the Preferred Reporting Items for Systematic Reviews and Meta-Analyses (PRISMA) statement [16] and the Cochrane Handbook [17]. Ethical approval was not required since this is a meta-analysis of published studies.

### Inclusion and exclusion criteria

All studies included in this meta-analysis met the following criteria: (1) published clinical RCT; (2) patients with a clear diagnosis of CTS, and the age, gender, and nationality were not limited; and (3) ESWT was used as an intervention measure, and CTS was used as a control measure, and complete comparison data between ESWT and CTS could be obtained.

Studies were excluded according to the following exclusion criteria: (1) CTS caused by trauma, fracture, tumor, infection, endocrine system disease, etc., or combined with diabetes, peripheral polyneuropathy, coagulation disorder, thrombosis, mental system disease, etc.; (2) patients who had received carpal tunnel surgery; (3) patients who had received oral hormones, non-steroidal anti-inflammatory drugs, and splint fixation before being enrolled; and (4) animal experiments.

### Search strategies

A systematic computer-based retrieval was performed on the literatures published before September 1, 2020, in PubMed, Embase, Cochrane Library, China National Knowledge Infrastructure database (CNKI), WanFang database, and Chinese Scientific Journal Database. The following search terms were used: “extracorporeal shock wave”, “local corticosteroid injection”, “injection”, “carpal tunnel syndrome” with the Boolean operators “AND” or “OR”. At the same time, we traced the references of the included literatures and the meta-analysis related to this research, screened, and evaluated the references to determine potential researches.

### Data extraction

Two researchers conducted the literature search, and strictly followed the inclusion and exclusion criteria for the preliminary screening and secondary screening of the literatures. After the screening, two independent researchers extracted data from the literatures that met the requirements and then the data were checked by the third researcher. Regarding any differences in the included literatures, consensus was reached through discussion among all researchers. The missing data in the literatures had been completed by contacting the authors by email. The main data extracted in this study include name of the first author, year of publication, sample size of the experimental group and the control group, gender ratio of patients, average age, intervention methods and treatment frequency, country, study design type, follow-up time, and outcome indicators. The extracted data had been reviewed to ensure accuracy.

### Quality assessment

This study used the Cochrane risk bias tool [18] for quality evaluation. This tool includes evaluations in seven aspects: random sequence generation, allocation hiding, blinding of participants and implementers, blinding of outcome evaluators, incomplete outcome data, selective reporting, and other biases. The risk of bias in each area is judged as low risk, high risk, or unknown risk. The quality of the studies was evaluated by two researchers.

### Data analysis

The Review Manager software (RevMan 5.3) was used for statistical analysis. Continuous variables were reported as mean difference (MD) and 95% confidence interval (CI), while dichotomy variables were reported as odds ratio (OR) and 95% CI. Statistical heterogeneity was judged by the combination of *Q* value statistics and *I*^2^ statistics. The larger the *I*^2^, the greater the heterogeneity. If there was heterogeneity in the study (*I*^2^ ≥ 50%), the random effects model was adopted; otherwise, the fixed effects model was adopted (*I*^2^ < 50%). The extracted data was input into the computer, reviewed, and independently analyzed by two researchers.

## Results

### Search result

**Fig. 1:**
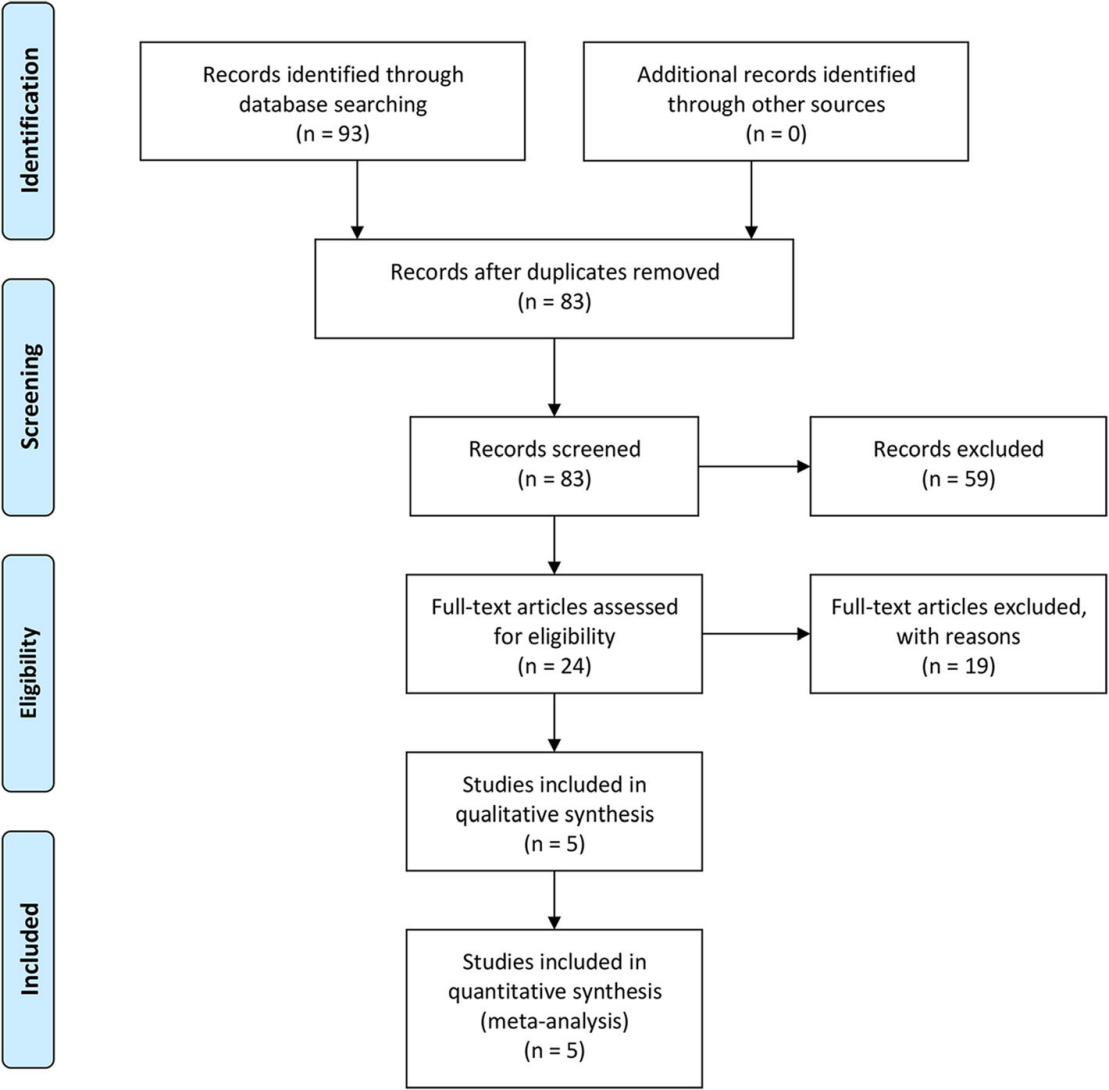
Flow diagram of the study selection process

A total of 93 related studies were confirmed from the electronic database. After deleting duplicate studies, 83 studies were obtained. After careful full-text evaluation of these studies according to the inclusion and exclusion criteria, 5 RCT studies [11–15] with 204 patients were included in the final comprehensive analysis. The literature screening flow diagram is shown in Fig. 1, and the basic characteristics of the included studies are shown in Table 1.

**Table 1:** Basic characteristics of the studies included

Author	Year	Sample size		Gender (female)		Mean age (years)		Intervention and treatment frequency		Country	Study design	Follow-up	Outcome assessment
		E	L	E	L	E	L	E	L				
Xu et al. [11]	2020	30	25	83.33%	84%	47.2 ± 1.86	46.9 ± 1.76	1000 shocks, 1.5 bar, 6 Hz once a week, 3 consecutive weeks	Betamethasone 40 mg one treatment	China	RCT	Week 3, 9, 12	VAS score, BQ score, sensory distal latency, motor distal latency, SNAP amplitude, CMAP amplitude
Atthakomol et al. [12]	2018	13	12	61.53%	91.67%	46 ± 9	53 ± 12	5000 shocks, 4 Bar 15 Hz one treatment	Triamcinolone acetonide 10 mg one treatment	Thailand	RCT	Week 1, 4, 12, 24	VAS score, BQ score, symptom severity score, functional score, sensory distal latency, motor distal latency, SNAP amplitude, CMAP amplitude
Sweilam et al .[13]	2019	25	28	84%	82.14%	37.6 ± 8.5	36.8 ± 8.8	2500 shocks, 2 bars, 10 Hz two treatments, 1 week apart	Triamcinolone acetonide 40 mg one treatment	Egypt	RCT	Week 2, 4	VAS score, BQ score, motor distal latency, CMAP amplitude, NCV of motor nerve
Seok and Kim [14]	2013	15	16	80%	87.5%	54.03 ± 19.47	49.67 ± 18.83	1000 shocks, 6 Hz one treatment	Triamcinolone acetonide 40 mg one treatment	Republic of Korea	RCT	Week 4, 12	VAS score, LSQ symptom severity score, LSQ functional status score, NCV of sensory nerve, SNAP amplitude, CMAP amplitude, sensory distal latency, motor distal latency
Tao et al. [15]	2018	20	20	65%	70%	54.9 ± 5.7	54.7 ± 5.6	Energy density 0.005–0.320 mJ/mm^2^, 0.5–20 Hz twice a week, 2 consecutive weeks	Compound betamethasone suspension 5 ml once a week, 2 consecutive weeks	China	RCT	Week 2	VAS score, BQ score, quality of life assessment-short form 36, motor distal latency, CMAP amplitude, NCV of sensory nerve

### Quality assessment

**Fig. 2:**
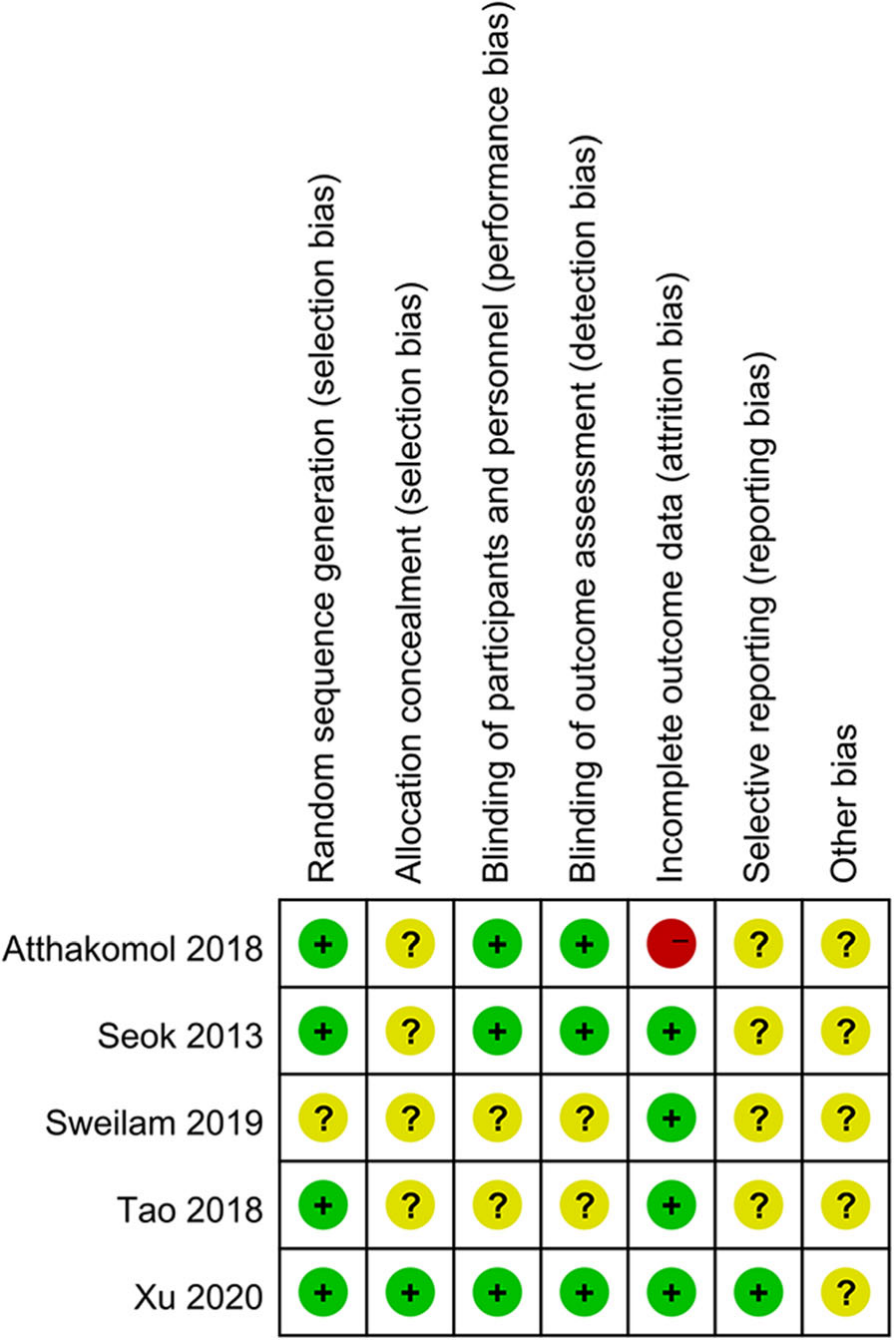
Risk of bias summary. Plus sign indicates low risk of bias. minus sign high risk ofbias, and question mark bias unclear

Among five analyzed RCTs, Xu et al.’s study used a computer to generate a list of random numbers and then generated a random sequence; patients were grouped using sealed envelopes, the test result evaluator was unaware of the grouping situation, there was no withdrawal or loss to follow-up, and the dataset was complete. The study of Atthakomol et al. used a random number generator to generate random sequences and further used envelopes for grouping; however, whether the envelopes were sealed was not stated. The evaluator of the test results was unaware of the results, and several patients withdrew or were lost to follow-up. In the study by Sweilam et al., the random sequence generation, allocation hiding, and blinding methodology were not provided. The study of Seok and Kim used random number generation software to generate random sequences. The allocation method was not specified; the test result evaluator was unaware of the results, and there was no withdrawal or loss to follow-up. Tao et al.’s study used a random number table method to generate random sequences. There was no withdrawal or loss to follow-up, but the remaining factors were not explained. As shown in Fig. 2.

### Comparison of the visual analog scale (VAS) scores for ESWT and LCI

**Fig. 3:**
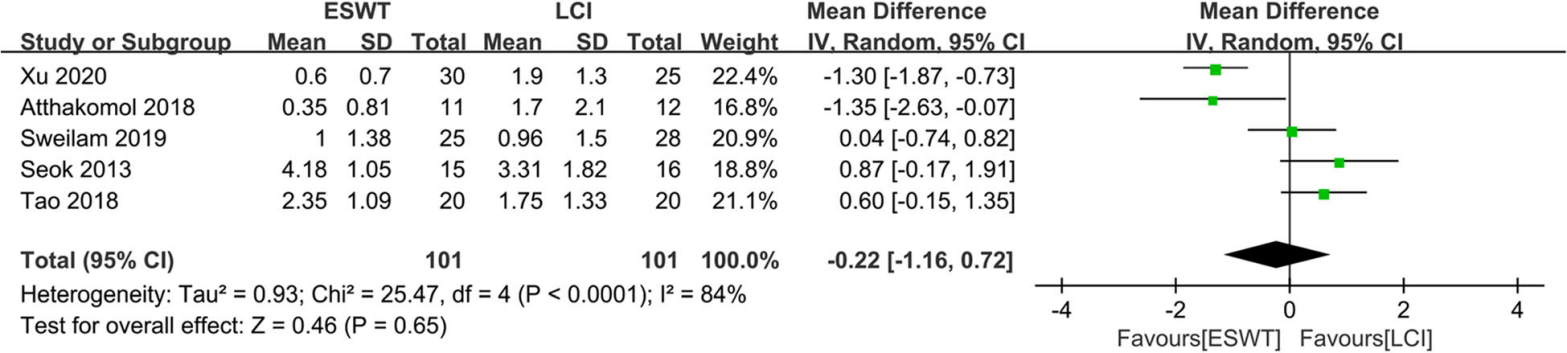
Forest plot showing the comparison of VAS score between ESWT and LCI for CTS

The VAS scores of ESWT and LCI were compared in five studies [11–15], including 202 patients (101 patients received ESWT treatment and 101 patients received LCI treatment), as shown in Fig. 3. The heterogeneity test showed that there was heterogeneity between the studies (*P* < 0.0001, *I*^*2*^ = 84%), so the random effects model was used to analyze the data of the five studies. The comprehensive results showed that the difference between the ESWT group and the LCI group was not statistically significant (MD − 0.22, 95%CI − 1.16 to 0.72, *P* = 0.65).

### Comparison of the Boston Carpal Tunnel Questionnaire (BQ) scores for ESWT and LCI

**Fig. 4:**
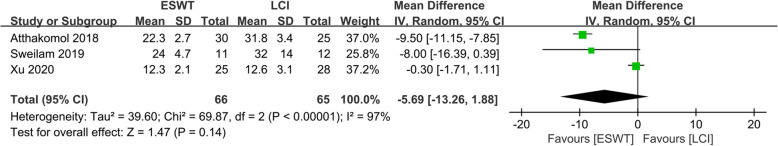
Forest plot showing the comparison of BQ score between ESWT and LCI for CTS

The BQ scores of ESWT and LCI were compared in three included studies [11–13], including 131 patients (66 patients received ESWT treatment and 65 patients received LCI treatment), as shown in Fig. 4. The heterogeneity test showed that there was heterogeneity between the studies (*P* < 0.00001, *I*^*2*^ = 97%), so the random effects model was used to analyze the data of three studies. The comprehensive results showed that the difference between the ESWT group and the LCI group was not statistically significant (MD − 5.69, 95%CI − 13.26 to 1.88, *P* = 0.14).

### Comparison of the sensory distal latency for ESWT and LCI

**Fig. 5:**
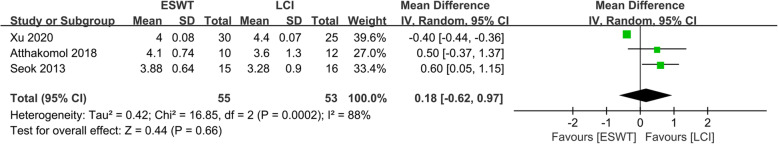
Forest plot showing the comparison of sensory distal latency between ESWT and LCI for CTS

The sensory distal latency of ESWT and LCI was compared in three included studies [11, 12, 14], including 103 patients (55 patients received ESWT treatment, 53 patients received LCI treatment), as shown in Fig. 5. The heterogeneity test showed that there was heterogeneity between the studies (*P =* 0.0002, *I*^*2*^ = 88%), so the random effects model was used to analyze the data of the three studies. The comprehensive results showed that the difference between the ESWT group and the LCI group was not statistically significant (MD 0.18, 95%CI − 0.62 to 0.97, *P* = 0.66).

### Comparison of the motor distal latency for ESWT and LCI

**Fig. 6:**
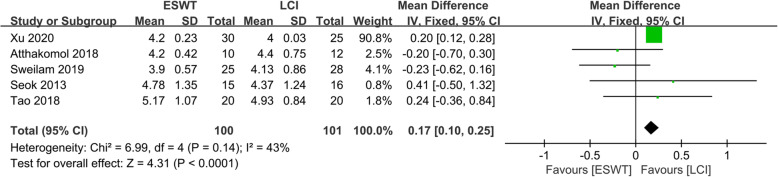
Forest plot showing the comparison of motor distal latency between ESWT and LCI for CTS

The motor distal latency of ESWT and LCI was compared in five included studies [11–15], including 201 patients (100 patients received ESWT treatment, 101 patients received LCI treatment), as shown in Fig. 6. The heterogeneity test showed that there was no heterogeneity between the studies (*P* = 0.14, *I*^*2*^ = 43%), so the fixed effects model was used to analyze the data of the five studies. The comprehensive results showed that the difference between the ESWT group and the LCI group was statistically significant (MD 0.17, 95% CI 0.10 to 0.25, *P* < 0.0001).

### Comparison of the compound muscle action potential (CMAP) amplitude for ESWT and LCI

**Fig. 7:**
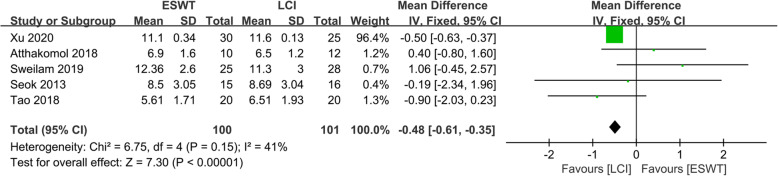
Forest plot showing the comparison of CMAP amplitude between ESWT and LCI for CTS

The CMAP amplitudes of ESWT and LCI were compared in five included studies [11–15], including 201 patients (100 patients received ESWT treatment and 101 patients received LCI treatment), as shown in Fig. 7. The heterogeneity test showed that there was no heterogeneity between the studies (*P* = 0.15, *I*^*2*^ = 41%), so the fixed effects model was used to analyze the data of the five studies. The comprehensive results showed that the difference between the ESWT group and the LCI group was statistically significant (MD − 0.48, 95%CI − 0.61 to − 0.35, *P* < 0.00001).

### Comparison of the sensory nerve action potential (SNAP) amplitude for ESWT and LCI

**Fig. 8:**
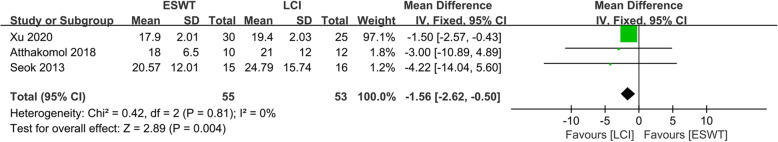
Forest plot showing the comparison of SNAP amplitude between ESWT and LCI for CTS

The SNAP amplitudes of ESWT and LCI were compared in three included studies [11, 12, 14], including 108 patients (55 patients received ESWT treatment, 53 patients received LCI treatment), as shown in Fig. 8. The heterogeneity test showed that there was no heterogeneity between the studies (*P* = 0.81, *I*^*2*^ = 0%), so the fixed effects model was used to analyze the data of the three studies. The comprehensive results showed that the difference between the ESWT group and the LCI group was statistically significant (MD − 1.56, 95%CI − 2.62 to − 0.50, *P* = 0.004).

### Comparison of the nerve conduction velocity (NCV) of sensory nerve for ESWT and LCI

**Fig. 9:**
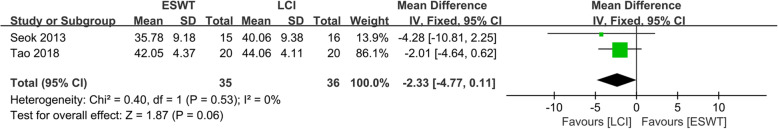
Forest plot showing the comparison of NCV of sensory nerve between ESWT and LCI for CTS

The NCV of sensory nerve of ESWT and LCI were compared in two included studies [14, 15], including 71 patients (35 patients received ESWT treatment, 36 patients received LCI treatment), as shown in Fig. 9. The heterogeneity test showed that there was no heterogeneity between the studies (*P* = 0.53, *I*^*2*^ = 0%), so the fixed effects model was used to analyze the data of the two studies. The comprehensive results showed that the difference between the ESWT group and the LCI group was not statistically significant (MD − 2.33, 95%CI − 4.77 to 0.11, *P* = 0.06).

### Publication bias

**Fig. 10:**
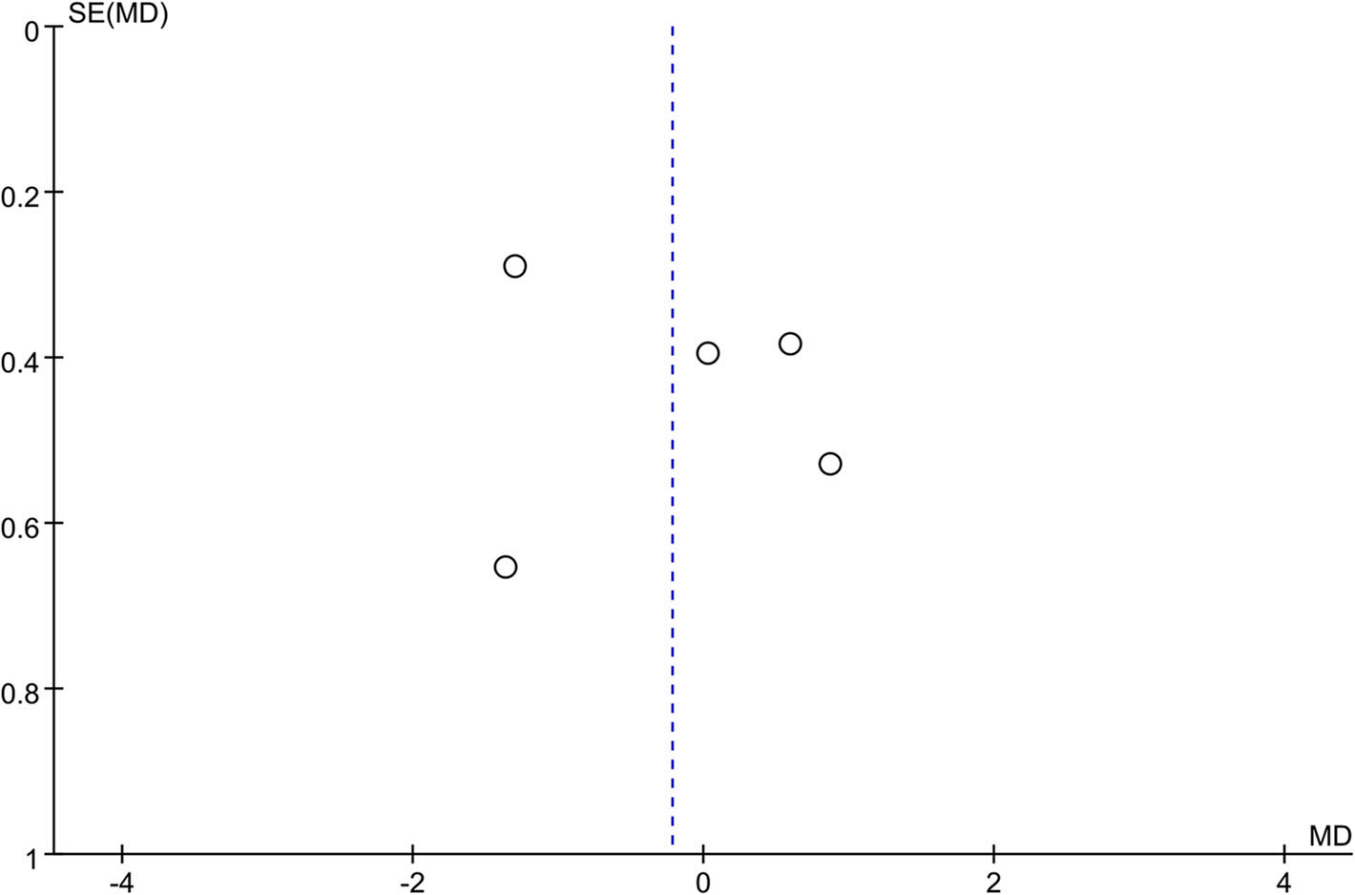
Funnel plot to detect publication bias for the studies

VAS score was the common outcome index of five RCT studies, and it was also the main indicator for CTS symptoms. Therefore, this outcome index was used to make a funnel plots to detect publication bias, as shown in Fig. 10. Visual inspection of the funnel plots showed symmetry, suggesting that there was no publication bias.

### Sensitivity analysis

For VAS score (*I*^*2*^ = 84%), BQ score (*I*^*2*^ = 97%) and sensory distal latency (*I*^*2*^ = 88%), the sensitivity was tested by eliminating the literature one by one, and found after excluding the study of Xu et al., the *I*^*2*^ and *P* values of the VAS score did not change much, while BQ score and sensory distal latency had no significant heterogeneity (*I*^*2*^ = 0%), and the differences between groups changed from non-statistically significant before elimination to statistically significant.

### GRADE evidence quality evaluation

The motor distal latency and CMAP amplitude of the two therapies were high-level evidence quality. VAS score, SNAP amplitude, and NCV of sensory nerve were moderate-level evidence quality. BQ score and sensory distal latency were very low-level evidence quality. It indicates that this result still needs to be further confirmed by higher quality RCTs, as shown in Table 2.

**Table 2:** Results of GRADE evidence evaluation

Outcome indicators	Risk of bias	Inconsistency	Indirectness	Imprecision	Publication bias	Upgrade conditions	Overall quality of evidence	Importance
VAS score [11–15]	None serious	Serious^a^	None serious	None serious	Undetected	None	⊕⊕⊕ Moderate	Important
BQ score [11–13]	None serious	Serious^a^	None serious	Serious^b^	Undetected	None	⊕ Very low	Important
Sensory distal latency [11, 12, 14]	None serious	Serious^a^	None serious	Serious^b^	Undetected	None	⊕ Very low	Important
Motor distal latency [11–15]	None serious	None serious	None serious	None serious	Undetected	None	⊕⊕⊕⊕ High	Important
CMAP amplitude [11–15]	None serious	None serious	None serious	None serious	Undetected	None	⊕⊕⊕⊕ High	Important
SNAP amplitude [11, 12, 14]	None serious	None serious	None serious	Serious^b^	Undetected	None	⊕⊕⊕ Moderate	Important
NCV of sensory nerve [14, 15]	None serious	None serious	None serious	Serious^b^	Undetected	None	⊕⊕⊕ Moderate	Important

^a^The heterogeneity test showed that there was high heterogeneity between the studies

^b^Small number of studies

## Discussion

In this study, we identified five RCT studies [11–15] involving 204 total patients to compare the clinical efficacy of ESWT and LCI on CTS. We evaluated seven outcome indicators, of which VAS score and BQ score were the main outcome indicators, while motor distal latency, sensory distal latency, CMAP amplitude, SNAP amplitude, and NCV of the sensory nerve were secondary outcome indicators. Our meta-analysis results showed that the results of the two therapies were not significantly different in terms of VAS score, BQ score, sensory distal latency, and NCV of the sensory nerve. There were significant differences between the results of the two therapies with respect to motor distal latency, CMAP amplitude, and SNAP amplitude.

Treatment of CTS usually involves splint fixation, LCI, and surgical release. The effectiveness of these methods has been clinically established [19]. The clinical basis of wrist splint fixation is that CTS symptoms appear to improve with rest and are aggravated with activity. The therapeutic effect comes from minimizing carpal tunnel pressure, but there is insufficient evidence for overall effectiveness [20]. Surgery release can quickly relieve any compression, but postoperative complications and delayed return to work may occur [21]. Moreover, some untreated patients will improve spontaneously without surgical intervention. If all CTS patients necessarily undergo carpal tunnel lysis, unnecessary surgeries may be performed [22]. LCI can effectively reduce inflammation and edema of the synovium and tendons, thereby reducing pressure on the median nerve, and can even stabilize the nerve membrane [23]. Its short-term efficacy is strongly supported, and it is therefore the preferred treatment for patients with mild to moderate CTS [24]. Shock waves are defined as a series of acoustic pulses with high pressure peaks, rapid pressure rises, and short durations; they are transient pressure disturbances that propagate in three-dimensional space [14]. ESWT is a noninvasive treatment method. It is produced in vitro and is focused on a specific part of the body. It usually uses fluid (water) and coupling gel as a conductive medium for transmission to biological tissues. Its clinical effects have also validated across multiple research studies [6–15].

In the investigated RCT [11–15], ESWT and LCI both showed good clinical efficacy in terms of pain relief, functional improvement, and electrophysiological parameter improvement, which is consistent with the conclusions of the above studies. The purpose of our investigation was to compare the clinical effects across the two therapies, which has been lacking to date. This study is the first meta-analysis to compare the effects of ESWT and LCI for the treatment of CTS. Our results showed that there was no significant difference in the VAS scores and BQ scores between the ESWT and LCI therapies. This shows that, despite the different mechanisms of action, the two therapies are equally effective in relieving pain and improving wrist joint function. LCI relieves pain and improves function by reducing inflammation and edema of the synovium and tendons, reducing nerve compression [23]. Although the mechanism of ESWT in CTS is not yet fully understood, its effects may stem from anti-inflammatory and nerve regeneration mechanisms [9]. Anti-inflammatory effects are seen with both ESWT and LCI, but the mechanism of action of ESWT is different from LCI. The anti-inflammatory effect of LCI is achieved by constricting blood vessels in the inflammation site, reducing capillary permeability, and stabilizing the lysosomal membrane, among other effects [23]. In contrast, ESWT stimulates the expression of structural NO synthase in soft tissues in response to pressure, resulting in increased physiologic levels of the powerful inflammation inhibitor NO [25–27]. Additional studies have suggested that the mechanism of pain relief may be caused by a decreased expression of calcitonin gene-related peptide in dorsal root ganglion neurons [28]. ESWT can also increase muscle sensitivity, which helps promote functional recovery [29]. The advantage of LCI is that as a conventional therapy, it is more widely used clinically and its efficacy is supported by more evidence; however, it is an invasive therapy. Due to potential complications and patient tolerance, the frequency and course of LCI treatment are limited. In contrast, ESWT is a noninvasive treatment. Its energy level, time of each treatment, treatment frequency and treatment course can be adjusted according to the patient’s condition. Patient tolerance is better, and cumulative clinical efficacy is easier to achieve [21]. ESWT has fewer complications and is safer, which is a major advantage of ESWT over LCI.

In terms of electrophysiological parameters, there was no significant difference between the two treatment groups with respect to NCV of the sensory nerve, but there were significant differences in motor distal latency, CMAP amplitude, and SNAP amplitude. The effect of LCI in shortening motor distal latency was stronger than that of ESWT, and ESWT was superior to LCI for improving SNAP amplitude and CMAP amplitude. Although both treatment methods improve nerve damage and nerve excitability, the mechanisms of the two therapies are different. LCI improves nerve damage by reducing inflammation and edema of the synovium and tendons, thereby reducing nerve compression and stabilizing the nerve membrane [23]. In contrast, ESWT can promote the regeneration of nerve fibers. Animal experiments have shown that ESWT can lead to angiogenesis, tissue repair, neurogenesis, and Schwann cell activation [29, 30]. ESWT can also increase the rate of axon regeneration. This phenomenon may include faster Wallerian degeneration, enhanced removal of degenerated axons, and a greater ability to regenerate damaged axons [31]. Several studies suggest that this is achieved through direct shock wave effects and indirect cavitation effects, which lead to hematoma formation and focal cell death [5]. It should be noted that most studies suggest that there is no significant relationship between CTS symptom severity score and electrodiagnosis results, and the impact of both intervention measures on electrodiagnosis results appears uncertain [12, 32–34]. The reason may be that electrodiagnosis can only detect large myelinated nerves. The function of small unmyelinated sensory nerves associated with CTS symptoms cannot be assessed by electrodiagnostic measurements. Therefore, neurogenic pain in CTS patients should be processed and treated independently of electrophysiologic data [33]. In the investigated studies, the follow-up time ranged from 1 to 24 weeks. We speculate that it may take longer follow-up observations to fully assess the recovery of the injured nerves and the regaining of complete function. Moreover, the specific parameters of ESWT, such as the number of shocks, energy size, frequency, pressure, and total treatment course, may also be factors that affect subsequent changes in electrophysiological parameters [5, 11]. This requires further research studies with longer follow-up times to fully evaluate.

None of the five studies we investigated showed significant complications. According to reports from other studies, LCI, as an invasive treatment, has more complications. LCI can adversely affect the function of tendon cells by reducing the synthesis of collagen and proteoglycans and ultimately reduce the mechanical strength of the tendon, leading to further degeneration. Corticosteroids or local anesthetics have also reportedly caused traumatic adventitia damage to peripheral nerve fibers or adventitia cells via hydrostatic pressure, axon and myelin degeneration, intrafascial cavities, tendon rupture, soft tissue atrophy, and accidental nerve damage [35–37]. To date, serious complications of ESWT treatment of CTS have not been reported in the literature [6–10, 12]. There may reportedly be short-term pain, skin redness, or small hematoma formation after ESWT treatment, but these resolve spontaneously [38].

### Limitation

Our meta-analysis has the following limitations. First, many reported studies did not meet the inclusion criteria. The sample size of the five RCT studies included is relatively small, and the quality of the studies is not sufficiently high, which may influence the overall research conclusions. Second, the included studies are heterogeneous. This may be due to differences in the various ESWT impact times, energy flow density, pressure, frequency, duration of action, and other parameters, as well as differences in LCI drug doses. In addition, the differences in treatment frequency and follow-up times likely also increase the heterogeneity. Third, the included studies did not have sufficiently lengthy follow-up times. The longest follow-up period was 24 weeks. The long-term efficacy, recurrence rate, and complications associated with the two therapies remains to be definitively investigated and compared [20, 39]. Finally, in the process of literature screening, it was found that the outcome indicators selected by each literature were quite different, and the outcome indicators shared by the five studies were few. We hope that the outcome indicators can be unified, so that the comparison results can be more convincing. It can more objectively and truly reflect the treatment effect and changes in the patient’s condition. Last but not the least, although our research results show efficacy of both ESWT and LCI, neither of these two therapies can directly relieve compression of the median nerve. For severely affected CTS patients, as well as patients whose symptoms have not improved significantly with treatment or have recurred after treatment, surgery may ultimately be required [11, 20].

## Conclusion

In summary, in terms of pain relief and function improvement, the effects of ESWT and LCI for the treatment of CTS are not significantly different. In terms of electrophysiological parameters, the two therapies each have their own advantages. LCI has a stronger effect on shortening motor distal latency; ESWT is superior to LCI in improving action potential amplitude. ESWT is a noninvasive treatment with fewer complications and greater patient safety. However, in light of the heterogeneity and limitations of the present study, these conclusions require further research for definitive conclusions to be drawn.

## Supplementary Information

**Additional file 1.** Search strategy.

## Data Availability

All data generated or analyzed during this study are included in this published article and its supplementary information files.

## Abbreviations

ESWT: Extracorporeal shock wave therapy
LCI: Local corticosteroid injection
CTS: Carpal tunnel syndrome
RCT: Randomized controlled trials
MD: Mean differences
OR: Odds ratios
CI: Confidence intervals
VAS: Visual analog scale
BQ: Boston Carpal Tunnel Questionnaire
NCV: Nerve conduction velocity
CMAP: Compound muscle action potential
SNAP: Sensory nerve action potential
