# Supplementary material for: Extracorporeal shock wave therapy versus local corticosteroid injection for the treatment of carpal tunnel syndrome: a meta-analysis
Source: J Orthop Surg Res. 2020 Nov 23;15:556. doi: 10.1186/s13018-020-02082-x (PMC7685634; doi:10.1186/s13018-020-02082-x)
Supplement: Supplementary file 1 — Additional file 1. Search strategy. [file 13018_2020_2082_MOESM1_ESM.docx]

***Search strategy***

***Pubmed***

*(extracorporeal shock wave) AND (local corticosteroid injection) AND (carpal tunnel syndrome) AND ("1966/01/01"[PDat]: "2020/09/01"[PDat])*

*Result: 4*

***Embase***

*#1 'extracorporeal shock wave' AND 'local corticosteroid injection' AND 'carpal tunnel syndrome'[1990-2020]/py*

*Result: 6*

***Cochrane library***

*4 Trials matching extracorporeal shock wave in All Text AND local corticosteroid injection in All Text AND* *carpal tunnel syndrome in All Text - with Cochrane Library publication date Between Jan 1990 and September 2020 (Word variations have been searched)*

*Result: 4*

***China National Knowledge Infrastructure databases (CNKI)***

*#1. 体外冲击波*

*#2. 注射*

*#3. 腕管综合征*

*#1 and #2 and #3*

*Result: 77*

**WanFang Database**

*#1. 体外冲击波*

*#2. 注射*

*#3. 腕管综合征*

*#1 and #2 and #3*

*Result: 1*

**Chinese Scientific Journal Database (VIP)**

*#1. 体外冲击波*

*#2. 注射*

*#3. 腕管综合征*

*#1 and #2 and #3*

*Result: 1*
